# A restatement of the natural science evidence base concerning neonicotinoid insecticides and insect pollinators

**DOI:** 10.1098/rspb.2014.0558

**Published:** 2014-07-07

**Authors:** H. Charles J. Godfray, Tjeerd Blacquière, Linda M. Field, Rosemary S. Hails, Gillian Petrokofsky, Simon G. Potts, Nigel E. Raine, Adam J. Vanbergen, Angela R. McLean

**Affiliations:** 1 Oxford Martin School, c/o Department of Zoology, University of Oxford, South Parks Road, Oxford OX1 3PS, UK; 2 Plant Research International, Wageningen University and Research, PO Box 16, 6700 AA Wageningen, The Netherlands; 3 Rothamsted Research, Harpenden, Herts AL5 2JQ, UK; 4 NERC Centre for Ecology and Hydrology, Crowmarsh Gifford, Wallingford OX10 8BB, UK; 5 School of Agriculture, Policy and Development, University of Reading, Reading, UK; 6 School of Biological Sciences, Royal Holloway University of London, Egham, Surrey TW20 0EX, UK; 7 NERC Centre for Ecology and Hydrology, Bush Estate, Penicuik, Edinburgh EH26 0QB, UK

**Keywords:** insecticides, neonicotinoids, pollination, honeybee, bumblebee, pollinator

## Abstract

There is evidence that in Europe and North America many species of pollinators are in decline, both in abundance and distribution. Although there is a long list of potential causes of this decline, there is concern that neonicotinoid insecticides, in particular through their use as seed treatments are, at least in part, responsible. This paper describes a project that set out to summarize the natural science evidence base relevant to neonicotinoid insecticides and insect pollinators in as policy-neutral terms as possible. A series of evidence statements are listed and categorized according to the nature of the underlying information. The evidence summary forms the appendix to this paper and an annotated bibliography is provided in the electronic supplementary material.

## 1. Introduction

Neonicotinoid insecticides are a highly effective tool to reduce crop yield losses owing to insect pests. Since their introduction in the 1990s, their use has expanded so that today they comprise about 30% by value of the global insecticide market [1]. They are commonly applied to crops as seed treatments, with the insecticide taken up systemically by the growing plant, so that it can be present in all plant parts, including nectar and pollen that bees and other pollinating insects collect and consume. Pollinators can potentially be exposed to neonicotinoids in other ways, for example through plant exudates, dust from planting machines and contamination of soil and water.

There is evidence that in Europe and North America many species of pollinators are in decline; both in abundance and distribution. There is a long list of potential causes for these declines, including parasites, disease, adverse weather and loss of habitat [2,3]. However, there has been particular concern about the impact on pollinators of the relatively recently introduced neonicotinoids and the European Union (EU) imposed a partial restriction on their use in December 2013. This decision has been criticized on the grounds that the benefits of neonicotinoid use outweigh any detriment they might cause.

The tension between the agricultural and environmental consequences of neonicotinoid use, and the recent EU restriction, has made this topic one of the most controversial involving science and policy. Here, we describe a project that aimed to provide a ‘restatement’ of the relevant natural science evidence base expressed in a succinct way that is comprehensible to non-expert readers. We have tried to be policy-neutral though are aware that complete neutrality is impossible. The evidence restatement forms appendix A to this paper and is accompanied in the electronic supplementary material by a detailed annotated bibliography that provides an entry into the technical literature. The restatement is divided into six sections: after a description of the methodology and the importance of pollinators and insecticides, successive sections consider evidence for exposure paths, laboratory evidence for lethal and sublethal effects, the occurrence of residues in pollinators and their products in the environment, experiments conducted in the field, and consequences for pollinators at colony and population levels.

Experiments to establish the effect of defined doses of insecticides upon individual pollinators are required by regulatory authorities and can be carried out under laboratory conditions. These laboratory studies have the strength of allowing carefully controlled experiments to be performed on individual insects subjected to well-defined exposure. However, because they are conducted under artificial conditions, it is hard to assess a number of processes that may be relevant in the field. For example, neonicotinoids may affect the sensitivity of insects to other stressors; pollinators may actively avoid food contaminated by insecticide and responses at the colony or population level may mitigate or exacerbate the loss or impairment of individual insects. Nevertheless, such experiments provide important information about the range of concentrations where death or sublethal effects are to be expected.

Purely observational surveys in the field are used to establish the levels of exposure that occur under normal use. A number of large surveys in different countries have measured neonicotinoid residues in wild-foraging honeybees and unmanaged pollinators, as well as in nectar, pollen, honey and wax within bee colonies. These data are heavily weighted towards honeybees, and long time series are seldom available.

Experiments in the field are used to establish the impact of different doses of insecticide on pollinator behaviour, mortality and colony performance. They may be conducted as part of the registration process or for general research. One class of experiment involves bees artificially exposed to neonicotinoids and then observed to forage in the field. These are designed to discover whether neonicotinoids affect the performance of individual pollinators (and where appropriate their colonies) under field conditions. The critical issue here is whether the experimental exposure to insecticides is representative of what pollinators are actually likely to experience. The second class of experiment involves placing bee colonies in the environment in situations where they are exposed to crops treated with neonicotinoids, with suitable controls. These are large, difficult experiments where the unit of replication is typically the field site and where there are potentially many confounding factors to be taken into consideration. So far only one such study has been concluded successfully. The statistical power of this type of experiment is likely to be constrained by the expense and logistics of high levels of replication.

To understand the consequences of changing neonicotinoid use, it is important to consider pollinator colony- and population-level processes, the likely effect on pollination ecosystem services, as well as how farmers might change their agronomic practices in response to restrictions on neonicotinoid use. While all these areas are currently being researched, there is at present a relatively limited evidence base to guide policy-makers.

## 2. Material and methods

The literature on pollinators and neonicotinoids was reviewed and a first draft evidence summary produced by a subset of the authors. At a workshop, all authors met to discuss the different evidence components and to assign to each a description of the nature of the evidence using a restricted set of terms. We considered several options to describe the nature of the evidence we summarize including the GRADE [4] system widely used in the medical sciences, or the restricted vocabulary used by the International Panel on Climate Change [5]. However, none precisely matched our needs and instead we used a scoring system based on one previously developed for another ‘restatement’ project concerning bovine tuberculosis [6]. The categories we used are:

— [D_ata_] a strong evidence base involving experimental studies or field *data* collection, with appropriate detailed statistical or other quantitative analysis;
— [E_xp_op_] a consensus of *expert opinion* extrapolating results from related ecological systems and well-established ecological principles;
— [S_upp_ev_] some *supporting evidence* but further work would improve the evidence base substantially; and
— [P_rojns_] *projections* based on the available evidence for which substantial uncertainty often exists that could affect outcomes.

These categories are explicitly not in rank order.

A revised evidence summary was produced and further debated electronically to produce a consensus draft. This was sent out to 34 stakeholders or stakeholder groups including scientists involved in pollinator research, representatives of the farming and agrochemical industries, non-governmental organizations concerned with the environment and conservation, and UK government departments and statutory bodies responsible for pollinator policy. The document was revised in the light of much helpful feedback. Though many groups were consulted, the project was conducted completely independently of any stakeholder and was funded by the Oxford Martin School (part of the University of Oxford).

## 3. Results

The summary of the natural science evidence base concerning neonicotinoid insecticides and insect pollinators is given in appendix A, with an annotated bibliography provided as the electronic supplementary material.

## 4. Discussion

The purpose of this project is not to conclude whether neonicotinoids are ‘safe’ or ‘dangerous’ but to try to help set out the existing evidence base. When neonicotinoids are used as seed dressing on crops visited by pollinators there is no doubt that these systemic insecticides are typically present in pollen and nectar and so bees and other pollinators can be exposed to them [7,8]. The concentrations in pollen and nectar are nearly always some way below those that would cause immediate death. The great problem is to understand whether the sublethal doses received by pollinators in the field lead to significant impairment in individual performance, and whether the cumulative effect on colonies and populations affects pollination in farmed and non-farmed landscapes and the viability of pollinator populations [3].

For this topic, the published literature is a small fraction of the evidence that has been collected. The process of registering a new insecticide requires the production of detailed environmental risk assessments (see http://eur-lex.europa.eu/LexUriServ/LexUriServ.do?uri=OJ:L:2013:093:0001:0084:EN:PDF and http://eur-lex.europa.eu/LexUriServ/LexUriServ.do?uri=OJ:L:2013:093:0085:0152:EN:PDF). These include substantial evidence on toxicity to non-target organisms (including honeybees) and a range of further studies that will, in some cases, escalate to full-scale field trials of toxicity. The data generated in such studies are not typically in the public domain, or only in a form summarized by the regulatory agencies, and hence we have not been able to include reference to them. There are understandable commercial reasons for the withholding of this information, though the chief reason is not that it contains proprietary intellectual property but that the information would be commercially advantageous to a competitor in registering the compound when it is out of licence. We wonder if registration rules might be amended to allow this type of data to be published, a clear public good, without disadvantaging companies that had invested in its collection.

If neonicotinoids are not available, then farmers will have to choose alternative pest-management strategies, alternative crops or accept greater losses. The impact upon pollinators of withdrawing neonicotinoids will be greatly influenced by such choices. Farmers' likely strategies when faced with restrictions on the use of neonicotinoids are being researched, but there is currently only limited evidence to guide policy-makers in what changes to expect. This is just one aspect of human behaviour, economics and other social science that may be relevant to questions about threats to pollinators. However, it was not the purpose of this review to summarize the social science literature in this area (the annotated bibliography provides an entry into this literature).

There is clear evidence of the great value of neonicotinoids in agriculture [1] as well as the importance of the ecosystem services provided to agriculture by managed and wild pollinators [9]. Pollinators also have intrinsic importance as components of natural biodiversity that cannot, or can only inexactly, be accorded economic value. In some cases, intelligent regulation of insecticide use can provide ‘win-wins’ that improve both agricultural and biodiversity outcomes but in other cases there will be trade-offs, both within and between different agricultural and environmental objectives. Different stakeholders will quite naturally differ in the weightings they attach to the variety of objectives affected by insecticide use, and there is no unique answer to the question of how best to regulate neonicotinoids, an issue that inevitably has both economic and political dimensions. But economic and political arguments need to be consistent with the natural science evidence base, even though the latter will always be less complete than desirable. We hope that our attempt to set out this evidence base in as policy-neutral a manner as possible will stimulate discussion within the science community about whether our assessments are fair and where investment most needs to be made to strengthen them. We hope it will also make the evidence base less contested and so help stakeholders from all perspectives develop coherant policy and policy recommendations.

## Appendix A. A restatement of the natural science evidence base concerning neonicotinoid insecticides and insect pollinators

For an annotated bibliography of the evidence supporting each statement, see the electronic supplementary material.

### (a) Introduction and aims

(1) Wild and managed insect pollinators play a critical role in the production of a variety of different foods (and in the case of honeybees also produce various ‘hive products’ of which the most important is honey) and are an important functional and cultural component of biodiversity. Insecticides are applied to crops to control insect pests and make a very important contribution to achieving high yields. Insecticides kill insects and thus clearly have both positive and negative effects on different aspects of food security and the environment. Concern has been expressed by a number of bodies that neonicotinoid insecticides may be harming pollinators and a partial restriction on their use in the EU came into force across all 28 member states in December 2013 (to be reviewed after 2 years). Other bodies have criticized this decision, arguing that the benefits of neonicotinoid use outweigh their costs.
(2) The aim here is to provide a succinct summary of the evidence base relevant to policy-making in this area as of April 2014. It also provides a consensus judgement by the authors on the nature of the different evidence components; a consensus arrived at using the studies listed in the annotated bibliography. We use the following descriptions, which explicitly are not a ranking, indicated by abbreviated codes. Statements are considered to be supported by:
  — [D_ata_] a strong evidence base involving experimental studies or field *data* collection, with appropriate detailed statistical or other quantitative analysis;
  — [E_xp_op_] a consensus of *expert opinion* extrapolating results from related ecological systems and well-established ecological principles;
  — [S_upp_ev_] some *supporting evidence* but further work would improve the evidence base substantially; and
  — [P_rojns_] *projections* based on the available evidence for which substantial uncertainty often exists that could affect outcomes.
(3) The review focuses on the natural science evidence relevant to pollinator policy in the EU but includes relevant data from other regions; its scope does not include evidence from social sciences and economics. The statements are based on the evidence in the peer-reviewed scientific literature, though the annotated bibliography also notes the existence of information in non-reviewed reports and industry studies.

### (b) Pollinators and neonicotinoid insecticides

(4) Insect pollinators are required to achieve optimum yield and quality for a number of important food crops. The most economically significant crops in the UK include oilseed rape (canola), soft fruits (strawberry, raspberry, etc.), top fruits (apple, pear, plum, etc.) and vegetables (courgettes, runner beans, tomato, etc.), whereas in continental Europe sunflower, peaches, melon and other crops are also important. Insect pollinators are important for both field crops and those grown under glass, though in their absence some crops can, to differing extents, be wind- or self-pollinated without the involvement of insects. Many plant species in pastureland and non-agricultural habitats require insect pollinators for successful reproduction [D_ata_].
(5) A lack of pollinators can reduce crop yields and quality [D_ata_], and there is some evidence that pollinator diversity can reduce the variance in pollination and hence improve crop yield stability [S_upp_ev_]. Where insect-pollinated crops are grown in glasshouses or ‘polytunnels’ the introduction of pollinators can be particularly important for both quality and quantity of yield [D_ata_]. There is emerging evidence for the potential of economically significant pollination deficits in some UK field crops in some years [S_upp_ev_], but data do not currently exist to determine whether observed changes in pollinator abundance and diversity (see para. 7) have affected the economic value of crop yields [E_xp_op_].
(6) Pollination may be carried out by wild or managed insects. The most important pollinators for crops include honeybees, which are native to Europe (their status in the British Isles is unclear [E_xp_op_]) but are now almost entirely managed, bumblebees, solitary bees and true flies (including hoverflies).^1^ Other pollinators such as butterflies and moths are not as important for crop pollination, particularly in northern temperate regions, but do pollinate wild plant species. Wild pollinators can be viewed as an element of natural capital^2^ that provides (with managed species) pollination, an ecosystem service of economic importance to society. Pollinators are also an important component of a nation's biodiversity [D_ata_].
(7) Data from volunteer recording schemes that record species presence (but not abundance or absence) have revealed changes in the diversity and distribution of pollinators. In Great Britain, The Netherlands and Belgium (where the best data exist) the average numbers of species of bumblebees, butterfly and moths, and solitary bees in different areas have declined since the 1950s [D_ata_]. There is some evidence of a recent slowdown in the rate of decline in species richness (for bumblebees in all three European countries) and also some increases (solitary bees in Great Britain and The Netherlands but not in Belgium where the decline continues) [D_ata_]. The data for hoverflies are more complex with species richness reported to have increased, decreased or remained unchanged depending on location and the geographical scale of the analysis. Long-term published data on abundance are only available for butterflies and moths and show reductions in abundance of many, but not all, species [D_ata_]. There are several potential (and non-exclusive) explanations for these observed changes in pollinator biodiversity with evidence suggesting habitat loss and alteration to be the most important causes of the decline [S_upp_ev_]. There is not a consensus on the reason(s) for recent slowdowns or reversals in the rates of species loss [E_xp_op_].
(8) Honeybees throughout Europe (and elsewhere) have been severely affected by the introduction of the *Varroa destructor* mite which both parasitizes bees and acts as a vector for a number of debilitating and paralytic honeybee viruses [D_ata_]. In addition, honeybee colony losses have increased in frequency across Europe and the USA because of overwintering mortality [D_ata_] which is thought to arise from multiple factors, including adverse weather, poor nutrition as well as parasites and disease [S_upp_ev_]. Some of these losses in the USA have been ascribed to a particular syndrome, colony collapse disorder, though its precise nature is debated [E_xp_op_]. Not all parts of the world have experienced recent increases in overwintering colony mortality [D_ata_].
(9) Neonicotinoids are a relatively new class of insecticide, introduced in the early 1990s. They target the nicotinic acetylcholine receptor (nAChR) with high affinity for insect receptors and low affinity for mammalian receptors and have relatively low (but not zero) mammalian and bird toxicity. They can be used as sprays, applied to soils as drenches or in granular form, introduced into irrigation water or injected into trees. However, they are most frequently (approx. 90% by volume in the UK) applied as seed treatments with the insecticide being taken up systemically by the growing plant. The convenience and cost-effectiveness of seed treatments, the development of resistance to other classes of insecticide by many insect pests, and restrictions on the use of other compounds, have resulted in neonicotinoids capturing 28.5% of the global insecticides market (2011; worth US$3.6B) and their wide use in Europe [D_ata_].
(10) Five neonicotinoids are approved for use in the EU: three from the *N-*nitroguanidine group—clothianidin, imidacloprid and thiamethoxam (metabolized to clothianidin in the plant, insect and environment); and two from the *N-*cyanoamidine group: thiacloprid and acetamiprid. Concern over their possible effects on pollinators has focused on the first three because they are the most used compounds, they have greater honeybee toxicity and they are used as seed treatments so can be present in the pollen and nectar of treated crops [D_ata_].
(11) In Europe (and elsewhere), environmental risk assessments of pesticides including all neonicotinoids are required before a product can come to market. A tiered approach has been adopted to ensure cost-effectiveness and proportionality. The tiers start with laboratory tests to determine hazard to a standard set of seven non-target organisms (including honeybees) and, if potential hazards are identified, may progress through more complex semi-field experiments and modelling to simulate exposure under different more realistic conditions, culminating with full-scale toxicity assessments to identify potential risks in the field. Field trials were conducted during the original environmental risk assessment process for neonicotinoids. Extensive data are often generated during the registration process but typically is not placed in the public domain, except in summary form [D_ata_].

### (c) Exposure of pollinators to neonicotinoid insecticides

(12) Neonicotinoids have been widely used in Europe as a seed treatment for oilseed rape, sunflowers, maize, potato, soya bean (and other crops such as cereals and beets not visited by pollinators).
  (a) A single treated oilseed rape seed is typically treated with approximately 35 μg neonicotinoids and a maize seed with 1.2 mg (see Endnote 3) [D_ata_].
  (b) Pollinators may be exposed to neonicotinoids applied as sprays. The use of *N-*nitroguanidine neonicotinoids at flowering time is restricted in most countries though acetamiprid and thiacloprid (from the less toxic *N-*cyanoamidine group) are sprayed on raspberries, fruit trees and oilseed rape at flowering time [D_ata_].
(13) The plant absorbs some of the insecticide from the seed treatment and as it grows the insecticide spreads to all plant parts including the nectar and pollen that bees and other pollinators collect and consume [D_ata_].
  (a) Estimates of the concentration of neonicotinoids in the pollen and nectar of seed-treated crops vary considerably with average maximum levels (from 20 published studies) of 1.9 (nectar) and 6.1 (pollen) ng g^−1^. Concentrations vary across crops and can be appreciably higher if neonicotinoids are applied as foliar sprays, soil drenches or through drip irrigation [D_ata_].
(14) Some plants secrete droplets of liquid (xylem sap) called guttation fluid at leaf tips or margins. High concentrations of neonicotinoids have been measured in the guttation fluid of seed-treated plants (up to 10^4^–10^5^ times that in nectar), especially when plants are young [D_ata_]. There has been concern that were pollinators to use guttation fluid as a source of water they would ingest highly toxic levels of insecticides. The available evidence does not suggest that pollinators collect guttation fluid containing neonicotinoids to any great extent, in part because it chiefly is present at times of the year when crops are unattractive to pollinators and other sources of water are present [E_xp_op_].
(15) Dust emitted from seed drilling machines can contain high concentrations of neonicotinoids; as well as being deposited on the soil, the dust can drift to contaminate neighbouring flowering crops and natural vegetation as well as surface waters. Sporadic incidents of mass honeybee mortality in several EU countries, the USA and Canada have been caused by dust from seed drilling machines [D_ata_].
  (a) Issues concerning dust chiefly involve the formulation of the insecticide, in particular, how it is made to ‘stick’ to the seed. EU and national regulations on formulation and seed drilling have been introduced to reduce the risks of these problems [D_ata_].
(16) Neonicotinoids introduced into the environment as seed treatments can affect soil insects and other invertebrates, effects considered in insecticide evaluation and registration. They persist in the environment with typical half-lives estimated to be of the order 15–300 days (with some longer estimates from laboratory studies and in the field under drought and freezing conditions). There is evidence that neonicotinoids can accumulate in soils when treated crops are grown repeatedly in the same field. Neonicotinoids can sometimes, but not always, be detected in weeds or in subsequent crops grown in the same soil, though when present the concentrations are considerably lower than in the target crop. Neonicotinoids have been detected in surface or groundwater around fields where they have been used as seed treatments [S_upp_ev_].
(17) Bees bring pollen and nectar (which in social bees is often extensively modified post-ingestion) to their hives or nests to feed their developing larvae [D_ata_] which thus may have different patterns of exposure and susceptibility compared with adults (see also para. 24) [S_upp_ev_].
(18) The risk of exposure to neonicotinoids for different pollinator species will be influenced by many aspects of their biology and ecology including body size, flower preference, whether they are a social species, and whether the time of year at which they are active (or in the case of social species experiencing rapid colony growth) coincides with the flowering of neonicotinoid-treated crops. There may also be differences in the physiological susceptibility of different pollinator species to neonicotinoids [E_xp_op_].
(19) The exposure of pollinators to neonicotinoids will be affected by the distribution of flowering crops in the landscape, the fraction that are treated with neonicotinoids, the length of time the treated crops are in flower, and the availability of alternative, suitable floral resources (including weeds and managed resources in floral strips, wildflower headlands, untreated crops, etc.) and whether they are contaminated with insecticide. Over multiple years the frequency of treated crops in agricultural rotations will affect long-term population exposure [E_xp_op_].
(20) The distance between treated fields and nest sites or honeybee hives will affect insect exposure to neonicotinoids [E_xp_op_].
  (a) Pollinators can forage over a large area: the maximum foraging distance for bumblebees is 2–3 km from the colony (though with considerable variation) and for honeybees 10–15 km (median distances are 1–6 km); some solitary bees may only forage a few hundred metres or less. Observed foraging distances are strongly influenced by the distribution of flowering plants [D_ata_].
(21) *Summary.* There are several proven pathways through which pollinators may be exposed to neonicotinoid insecticides applied as seed treatments (or in other ways). Quantitative information about the extent and significance of these different routes in the published literature is poor [E_xp_op_].

### (d) Laboratory studies of lethal and sublethal effects of neonicotinoids

(22) Estimates of LD_50_s (see Endnote 4) for different neonicotinoid-pollinator combinations are available, although a majority of the studies have considered only the honeybee [D_ata._].
  (a) The acute oral LD_50_s for the major neonicotinoids have been estimated (by EFSA^5^) to be 3.7 ng per honeybee for imidacloprid, 3.8 ng per honeybee for clothianidin and 5.0 ng per honeybee for thiamethoxam (these estimates are used in the calculations below). A meta-analysis of 14 studies of imidacloprid estimated an LD_50_ of 4.5 ng per honeybee (95% confidence limits 3.9–5.2 ng) [D_ata_].
  (b) Equivalent acute contact LD_50_s have been estimated (by EFSA) to be 81 ng per honeybee for imidacloprid, 44 ng per honeybee for clothianidin and 24 ng per honeybee for thiamethoxam [D_ata_].
  (c) There is considerable variation among LD_50_s measured across different bee species, and this is influenced by type of neonicotinoid and mode of application [D_ata_]. This complicates simple comparison with honeybee data [E_xp_op_].
  (d) A honeybee, returning to the hive after foraging, typically carries 25–40 mg nectar or 10–30 mg pollen. If nectar or pollen is contaminated with insecticide at the concentrations described in Para. 13a, then these loads will contain approximately 0.06 ng (nectar) or 0.12 ng (pollen) of insecticide. Depending on the type of neonicotinoid this is 1–3% of the LD_50_ acute oral dose (though note that none of the pollen and hardly any of the nectar is metabolized by the forager). A colony of 10 000 workers was observed to store 750 g of pollen in four days. If all the pollen was similarly contaminated this equates to 8–11% of the acute oral LD_50_ [P_rojns_].
  (e) Maximum pollen consumption is found among nursing honeybees that can consume 7.2 mg d^–1^. If the pollen contains 6.1 ng g^−1^ neonicotinoid the daily intake is 0.044 ng or, depending on the compound, 0.8–1.1% of the acute oral toxicity LD_50_. Maximum nectar consumption is found among nectar-foraging honeybees and can be 32–128 mg d^−1^. If nectar contains 1.9 ng g^−1^ neonicotinoid the daily intake is 0.061–0.243 ng, or 1.2–6.7% of the LD_50_ acute oral [P_rojns_].
  (f) Honeybee colonies collect pollen and nectar from multiple sources, which dilutes the effects of foraging on neonicotinoid-treated crops [D_ata_]. For this reason and because they are based on the average maximum neonicotinoid concentrations in Para. 13a, the calculations in subparagraphs d and e above should be viewed as a worst-case scenario [E_xp_op_].
(23) Prolonged exposure of pollinators in the laboratory to doses of neonicotinoids that do not cause immediate death can reduce longevity (chronic toxicity). Because chronic effects can be estimated in many different ways, comparisons are harder than for acute toxicity [D_ata_].
  (a) For honeybees and bumblebees, chronic lethal effects have typically been reported when bees are fed diets containing 10–20 ng g^−1^ neonicotinoid over 10–20 days, although some studies with higher doses have not observed such effects [D_ata_].
  (b) These neonicotinoid concentrations are higher than the worst-case assumptions of maximum insecticide consumption in para. 22e [P_rojns_].
(24) Effects of neonicotinoids on adult pollinators have been detected in the laboratory at doses substantially below those that cause death. At the lowest doses responses involve metabolic changes (for example, in acetylcholinesterase activity) and subtle neurological and behavioural responses. As doses increase (including concentrations in food similar to that observed in the nectar and pollen of treated crops) olfactory learning, memory and feeding behaviour can be affected, though there is considerable variability in the results reported in different studies. When doses approach lethal concentrations substantial neurological and locomotory impairment can occur [D_ata_].
  (a) The majority of studies have involved honeybees; where comparisons of honeybees with bumblebees and solitary bees have been made differences are frequently observed, although these depend on species, assay and type of neonicotinoid and general patterns are difficult to discern [S_upp_ev_].
  (b) There has been debate in the literature as to the extent that neonicotinoids accumulate in pollinators; recent studies have suggested that bees have a substantial capacity to extrude neonicotinoids from cells and tissue (honeybees were estimated to clear 2 ng d^−1^ imidacloprid from their body—approximately 50% of oral LD_50_—and larger bumblebees 7 ng d^−1^) [D_ata_].
(25) Sublethal effects on larval development and colony productivity have been identified in the laboratory.
  (a) Delayed larval and pupal developments have been observed in honeybees though at neonicotinoid concentrations higher than those expected to occur in the field [D_ata_].
  (b) Increases in development time, and reductions in worker egg laying, worker production, worker longevity and male and new queen (gyne) production have been observed in bumblebee colonies when food is provided containing concentrations of neonicotinoids towards the high end of those observed in nectar and pollen in treated crops in the field. Similar results have been found for larval development and reproductive output in solitary bees [S_upp_ev_].
(26) Stressed pollinators tend to be more susceptible to neonicotinoids (and vice versa), although data are largely restricted to honeybees [S_upp_ev_].
  (a) Honeybees stressed by disease are more susceptible (lethal and sublethal effects occur at lower doses) to neonicotinoids, whereas in bumblebees synergistic effects of neonicotinoids and parasites on queen longevity, but not other colony parameters, have been observed. Neonicotinoids can modulate insect innate immunity negatively affecting anti-viral and other defences [D_ata_].
  (b) Laboratory molecular biological studies show a potential for the presence of other pesticides (targeted at fungi and *Varroa*) to exacerbate the effects of neonicotinoids though there is limited evidence for such effects from studies with live insects [S_upp_ev_].
  (c) It is likely that pollinators exposed to poorer diets are more susceptible to neonicotinoids (and other stressors) [E_xp_op_].
(27) In interpreting these laboratory results, the following issues need to be considered:
  (a) There is extensive information on the acute lethality of major neonicotinoids in honeybees, but data on other effects, on other pollinators and with the full range of neonicotinoids, are more limited [E_xp_op_].
  (b) Stress affects insect responses to neonicotinoids and laboratory conditions may be more or less stressful than in the field, an effect that is probably pollinator-species specific and rarely directly assessed in experiments [E_xp_op_].
  (c) Laboratory experiments normally involve feeding pollinators with sugar solution or mixed pollen which may affect insects differently to naturally collected food [E_xp_op_].
  (d) Chronic and sublethal effects will depend on the pattern of dietary consumption and the rate at which ingested neonicotinoids are cleared from the body [E_xp_op_]. In addition, neonicotinoids can act as anti-feedants and hence may affect pollinators through reduced food intake, though typically at concentrations higher than expected in the field. How insecticide treated food is presented to pollinators in laboratory experiments, and whether the insects have access to alternative foods, will thus influence the observed responses [S_upp_ev_].
  (e) It is challenging to study the impacts of neonicotinoids on entire colonies in the laboratory (particularly for honeybees). As a result, the majority of laboratory studies examine effects on individual bees or queen-less groups (often referred to as micro-colonies in bumblebee studies). These results need careful interpretation when assessing how they might translate to whole colony impacts for social bees in the field [E_xp_op_].
(28) *Summary.* The strengths of laboratory studies are that they allow carefully controlled experiments to be performed on individual insects subjected to well-defined exposure. The weaknesses are that they are conducted under very artificial conditions (which may affect tolerance to external stress), any avoidance response by the insect is limited and hence the exposure dose and form is determined solely by the experimenter, and responses at the colony or population level are both difficult to study and to extrapolate to the field. Nevertheless, they provide important information about the range of concentrations where death or sublethal effects may be expected to occur [E_xp_op_].

### (e) Neonicotinoid residues observed in pollinators in the field

(29) Nectar and pollen collected from bees constrained to feed on treated crops have similar insecticide concentrations to those found in samples taken from the plant [D_ata_].
(30) There have been few surveys of pesticide and metabolite levels in honeybees in the field. Two studies in Belgium (sample size, *n* = 48 and 99) and one in the USA (*n* = 140) found no honeybees with residues, while a survey in France conducted in 2002–2005 (*n* = 187) detected imidacloprid in 11% of honeybees (at concentrations of 0.03–1.0 ng per bee) [D_ata_]. We are aware of no data on other pollinators [E_xp_op_].
(31) Insecticide residues are more likely to be found in nectar and pollen collected by honeybees and in honey than in the insects themselves. Thus, the French study that found imidacloprid residues in 11% of the bees sampled also found residues in 22% of honey samples and 40% of pollen samples (mean and range: 0.9, 0.2–5.7 ng g^−1^). Some large surveys (e.g. a Spanish study with *n* = 1021) found no contaminated pollen; a German study that surveyed hives (*n* = 215) after oilseed rape flowering found low incidence of those neonicotinoids used in seed treatments (though higher incidence of thiacloprid); an American study found imidacloprid in 3% of pollen (*n* = 350) and 1% of wax samples (*n* = 208) [D_ata_].
(32) *Summary.* Neonicotinoids can be detected in wild pollinators as well as honeybee and bumblebee colonies but data are relatively few and restricted to a limited number of species. Studies to date have found low levels of residues in surveys of honeybees and honeybee products. Observed residues in bees and the products they collect will depend critically on details of spatial and temporal sampling relative to crop treatment and flowering [E_xp_op_].

### (f) Experiments conducted in the field

(33) This section discusses recent studies that have explored the consequences of providing bee colonies placed in the field with food containing insecticide, as well as experiments where the performance of colonies placed adjacent to fields treated or not treated with neonicotinoids are compared. Some earlier studies with limited statistical power are listed in the annotated bibliography [E_xp_op_].
(34) Schneider *et al.* 2012 [10]*.* Individual honeybees were given single sublethal doses of imidacloprid or clothianidin and their foraging behaviour was monitored. Reductions in foraging activity and longer time foraging flights were not observed at field-relevant doses although negative effects were seen at doses greater or equal to 0.5 ng per bee (clothianidin) or 1.5 ng per bee (imidacloprid) [D_ata_].
  (a) These doses are higher than those likely to be encountered by honeybees foraging on nectar from treated plants (see calculations in para. 22e) [E_xp_op_]
(35) Henry *et al.* 2012 [11]*.* Honeybees fed a single high dose of thiamethoxam (1.34 ng, equivalent to 27% of the LD_50_) and then released away from the hive were significantly less likely to return successfully than controls. The return rate depended on the local landscape structure and the extent of the honeybees' experience of the landscape. The failure to return per trip was estimated to be up to twice the expected background daily mortality [D_ata_].
  (a) The rate of forager loss per trip (15%) was analysed as if it were excess daily mortality but as foraging honeybees make 10–30 trips per day real loss rates would be very much higher, reflecting the high dose of insecticide used in the experiment (see para. 22e for calculation of likely field doses) [E_xp_op_].
  (b) Assuming honeybees were exposed every day to this dose rate (much higher than expected from observed residues in pollen and nectar), mathematical modelling of colony development predicted severe decline within a season though this conclusion depends critically on poorly understood aspects of honeybee colony dynamics [P_rojns_].
(36) Whitehorn *et al.* 2012 [12]*.* Bumblebee (*Bombus terrestris*) colonies fed exclusively on imidacloprid-treated sugar water (at two concentrations: 0.7 or 1.4 ng g^−1^) and pollen (either 6 or 12 ng g^−1^) for two weeks in the laboratory before being placed in the field (for six weeks) showed reductions in growth rate and queen production. A subsequent study [13] using the same concentrations of imidacloprid found the bumblebees' capacity to forage for pollen (but not nectar) was impaired [D_ata_].
  (a) The concentrations of insecticide are at the high end of those observed in the nectar and pollen of treated plants (Para. 13a) and are likely to be greater than most bees will receive in the field because alternative food sources were not available [E_xp_op_].
(37) Gill *et al.* 2012 [14]. Bumblebee (*B. terrestris*) colonies given access to sugar water containing imidacloprid (10 ng g^−1^) and allowed to forage for pollen and nectar in the field grew more slowly than controls; individual foragers from imidacloprid-treated colonies were less successful at collecting pollen, and treated colonies sent out more workers to forage and lost more foragers, compared to controls. Combined exposure to imidacloprid and a second pesticide of a different class (a pyrethroid) tended to reduce further colony performance and increase the chances of colony failure [D_ata_].
  (a) The concentration of insecticide in the sugar water is within the range observed in nectar in the field but considerably higher than the average (1.9 ng g^−1^; Para. 13a). The actual amount of imidacloprid consumed by individual bumblebees was not measured but will be diluted through foraging from other sources (no pollen was provided). Although it is difficult to make precise comparisons, the pyrethroid concentrations used were towards the upper end of recommended application rates for field or fruit crops [E_xp_op_].
(38) Thompson *et al.* 2013 [15]. Bumblebee (*B. terrestris*) colonies were placed adjacent to single oilseed rape fields grown from seeds that were treated with clothianidin, imidacloprid or had no insecticidal seed treatment. No relationship between the oilseed rape treatment and insecticide residues was observed, presumably because the bees were foraging over spatial scales larger than a field. Insecticide residues varied among colonies and the authors reported no evidence of a correlation with colony performance [D_ata_].
  (a) The experimental design, in particular the lack of replication at field level and absence of a clear effect of treatment, allows only limited inference about the effects of neonicotinoids in the field [E_xp_op_].
(39) Pilling *et al.* 2013 [16]. Over a 4 year period, honeybee colonies (six per 2 ha field) were placed beside thiamethoxam-treated or control fields of maize (three replicates) or oilseed rape (two replicates) for between 5 and 8 days (first 3 years) or 19 and 23 days (fourth year) to coincide with the crop flowering period (at other times the colonies were kept in woodland presumed to have no local exposure to insecticides). Honeybees from treatment hives had higher concentrations of insecticide residues, but no differences in multiple measures of colony performance or overwintering survival were observed [D_ata_].
  (a) Levels of replication precluded formal statistical analysis though the lack of any differences between treatment and control was reasonably consistent across field sites [E_xp_op_].
(40) *Summary*. The experiments described in Paras. 33–37 involve bees artificially exposed to neonicotinoids and observed to forage in the field. They show the potential for neonicotinoids to affect the performance of individual pollinators and pollinator colonies in the field. The main issue for their interpretation is the extent to which the doses received by the bees are representative of what they will receive under normal use of neonicotinoids in the field. It appears that most studies have used concentrations at the high end of those expected in the field. The experiments described in Paras. 38 and 39 are true field experiments in the sense that the treatments involve the normal use of neonicotinoids, though only the Pilling *et al.* [16] study was successfully concluded and found no effects of neonicotinoids, but with limited statistical power to detect differences [E_xp_op_].

### (g) Consequences of neonicotinoid use

(41) At the colony or population level, there may be processes that can compensate for the deaths of individual insects which would mitigate the potential effects of mortality caused by neonicotinoid insecticides. Thus, the deaths of individual pollinators may not lead to a simple proportionate decrease in the overall numbers of that pollinator species. In the case of rare species, extra mortality caused by insecticides could lead to a threshold population density being crossed below which the species declines to extinction, hence magnifying their effects. However, there is a weak evidence base to help understand the presence and magnitude of these effects in the field. Models of honeybee and bumblebee colony dynamics, as well as population-level models of all pollinators, are important tools to explore these effects [E_xp_op_].
(42) There is evidence that some crops do not always receive sufficient pollination [D_ata_], and further limited evidence that this has increased in recent decades [S_upp_ev_]; but the information available does not allow us to determine whether or not this has been influenced by the increased use of neonicotinoids [E_xp_op_]. Whether pollination deficits in wild plants have increased is not known [E_xp_op_].
(43) Declines in the populations of many insect species in general and pollinators in particular have been observed (para. 7) although the decline in bees predate by some decades the introduction of neonicotinoid insecticides, and there is some evidence of a recent abatement in the rate of decline for some groups [D_ata_]. Habitat alteration (especially in farmland) is widely considered to be the most important factor responsible. The evidence available does not allow us to say whether neonicotinoid use has had an effect on these trends since their introduction [E_xp_op_].
(44) There have been marked increases in overwintering mortality of managed honeybee populations in recent decades (para. 8) [D_ata_]. It has been suggested that insecticides (particularly neonicotinoids) may be wholly or partly responsible. The weak evidence base cannot at present resolve this question although honeybee declines began before the wide use of neonicotinoids and there is poor geographical correlation between neonicotinoid use and honeybee decline [E_xp_op_]. Two studies using different structured methodologies have explored this question.
  (a) Cresswell *et al.* 2012 [17]. Used ‘Hill's epidemiological “causality criteria”’ and concluded that the evidence base did not currently support a role for dietary neonicotinoids in honeybee decline but that this conclusion should be seen as provisional [E_xp_op_].
  (b) Staveley *et al.* 2014 [18]. Used ‘causal analysis’ methodology and concluded that neonicotinoids were ‘unlikely’ to be the sole cause of honeybee decline but could be a contributing factor [E_xp_op_].
(45) Neonicotinoids are efficient plant protection compounds and if their use is restricted farmers may switch to other pest-management strategies (for example, different insecticides applied in different ways or non-chemical control measures) that may have effects on pollinator populations that could overall be more or less damaging than neonicotinoids. Alternatively, they may choose not to grow the crops concerned, which will reduce exposure of pollinators to neonicotinoids but also reduce the total flowers available to pollinators [E_xp_op_].
(46) *Summary*. To understand the consequences of changing neonicotinoid use, it is important to consider pollinator colony-level and population processes, the likely effect on pollination ecosystem services, as well as how farmers might change their agronomic practices in response to restrictions on neonicotinoid use. While all these areas are currently being researched there is at present a limited evidence base to guide policy-makers [E_xp_op_].

## References

[1] Jeschke P Nauen R Schindler M Elbert A 2011 Overview of the status and global strategy for neonicotinoids. J. Agric. Food Chem. 59, 2897–2908. (10.1021/jf101303g) 20565065

[2] Potts SG Biesmeijer JC Kremen C Neumann P Schweiger O Kunin WE 2010 Global pollinator declines: trends, impacts and drivers. Trends Ecol. Evol. 25, 345–353. (10.1016/j.tree.2010.01.007) 20188434

[3] Vanbergen AJ, Insect Pollinators Initiative 2013 Threats to an ecosystem service: pressures on pollinators. Front. Ecol. Environ. 11, 251–259. (10.1890/120126)

[4] Guyatt GH Oxman AD Vist G Kunz R Falck-Ytter Y Alonso-Coello P Schünemann HJ 2008 GRADE: an emerging consensus on rating quality of evidence and strength of recommendations. Br. Med. J. 336, 924–926. (10.1136/bmj.39489.470347.AD) 18436948 PMC2335261

[5] IPCC. 2007 Fourth Assessment Report of the Intergovernmental Panel on Climate Change (Contribution of Working Group II). Cambridge, UK: Cambridge University Press.

[6] Godfray HCJ 2013 A restatement of the natural science evidence base relevant to the control of bovine tuberculosis in Great Britain. Proc. R. Soc. B 280, 20131634 (10.1098/rspb.2013.1634). PMC3757986 23926157

[7] Blacquière T Smagghe G Van Gestel CAM Mommaerts V 2012 Neonicotinoids in bees: A review on concentrations, side-effects and risk assessment. Ecotoxicology 21, 973–992. (10.1007/s10646-012-0863-x) 22350105 PMC3338325

[8] Goulson D 2013 An overview of the environmental risks posed by neonicotinoid insecticides. J. Appl. Ecol. 50, 977–987. (10.1111/1365-2664.12111)

[9] Klein AM Vaissière BE Cane JH Steffan-Dewenter I Cunningham SA Kremen C Tscharntke T 2007 Importance of pollinators in changing landscapes for world crops. Proc. R. Soc. B 274, 303–313. (10.1098/rspb.2006.3721) PMC1702377 17164193

[10] Schneider CW Tautz J Grünewald B Fuchs S 2012 RFID tracking of sublethal effects of two neonicotinoid insecticides on the foraging behavior of *Apis mellifera*. PLoS ONE 7, e30023 (10.1371/journal.pone.0030023) 22253863 PMC3256199

[11] Henry M Béguin M Requier F Rollin O Odoux JF Aupinel P Aptel J Tchamitchian S Decourtye A 2012 A common pesticide decreases foraging success and survival in honey bees. Science 336, 348–350. (10.1126/science.1215039) 22461498

[12] Whitehorn PR O'Connor S Wackers FL Goulson D 2012 Neonicotinoid pesticide reduces bumble bee colony growth and queen production. Science 336, 351–352. (10.1126/science.1215025) 22461500

[13] Feltham H Park K Goulson D In press Field realistic doses of pesticide imidacloprid reduce bumblebee pollen foraging efficiency. Ecotoxicology 23, 317–323. (10.1007/s10646-014-1189-7) 24448674

[14] Gill RJ Ramos-Rodriguez O Raine NE 2012 Combined pesticide exposure severely affects individual-and colony-level traits in bees. Nature 491, 105–108. (10.1038/nature11585) 23086150 PMC3495159

[15] Thompson H Harrington P Wilkins W Pietravalle S Sweet D Jones A 2013 Effects of neonicotinoid seed treatments on bumble bee colonies under field conditions. See http://www.fera.co.uk/ccss/documents/defraBumbleBeeReportPS2371V4a.pdf.

[16] Pilling E Campbell P Coulson M Ruddle N Tornier I 2013 A four-year field program investigating long-term effects of repeated exposure of honey bee colonies to flowering crops treated with thiamethoxam. PLoS ONE 8, e77193. (10.1371/journal.pone.0077193) PMC3806756 24194871

[17] Cresswell JE Desneux N van Engelsdorp D 2012 Dietary traces of neonicotinoid pesticides as a cause of population declines in honey bees: an evaluation by Hill's epidemiological criteria. Pest Manag. Sci. 68, 819–827. (10.1002/ps.3290) 22488890

[18] Staveley JP Law SA Fairbrother A Menzie CA 2014 A causal analysis of observed declines in managed honey bees (*Apis mellifera*). Hum. Ecol. Risk Assess. 20, 566–591. (10.1080/10807039.2013.831263) 24363549 PMC3869053

